# A cutaneous lesion mimicking pacemaker infection

**DOI:** 10.1002/joa3.12819

**Published:** 2023-01-22

**Authors:** Fabiola Schafer, Lilia Antonio, Andrés Diaz, Rodrigo Miranda

**Affiliations:** ^1^ Department of Medical Specialties, School of Medicine Universidad de La Frontera Temuco Chile; ^2^ Department of Pathological Anatomy, School of Medicine Universidad de La Frontera Temuco Chile; ^3^ Department of Surgery, Traumatology & Anesthesia, School of Medicine Universidad de La Frontera Temuco Chile; ^4^ Department of Internal Medicine, School of Medicine Universidad de La Frontera Temuco Chile

**Keywords:** epidermoid inclusion cyst, infection, pacemaker, skin biopsy, ultrasound scan

A 69‐year‐old woman with a past medical history of 5‐year‐old dual‐chamber pacemaker implantation because of symptomatic sick sinus syndrome presented with swelling, erythema, and pain at the upper part of the pacemaker pocket (Figure 1A). Physical examination revealed a swollen, erythematous and painful skin lesion. She was afebrile without signs of systemic infection. The inflammatory skin lesion was over the site where the pacemaker leads run from the pulse generator to the subclavian vein insertion. Pacemaker leads were easily palpable under the skin lesion, so infection of them was suspected. She was treated with cefadroxil for 14 days with poor response. Indeed, the lesion changed to a violaceous color and increased in size along with a superficial ulceration and fluid discharge (Figure 1B). A surgical revision was indicated. Laboratory tests, chest x‐ray, and echocardiogram were all within normal limits. An ultrasound scan of the skin lesion showed a hypoechoic and heterogeneous nodule with well‐defined borders with no internal vascularity located in the dermis and subcutaneous tissue without compromise of deeper tissue and pacemaker pocket (Figure 1C). Fluoroscopy showed that the skin lesion was over the upper part of the pocket where the leads run (Figure 1D). Excision of the skin lesion was performed and revision of the area did not show compromise of the pacemaker leads. Skin biopsy informed a cutaneous fistula due to epidermal inclusion cyst (EIC; Figure 2A,B). The patient was treated with 10 days of oral flucloxacillin with complete clinical resolution. A close follow‐up has shown complete remission and no signs of infection after cessation of the antibiotic treatment.

**FIGURE 1:**
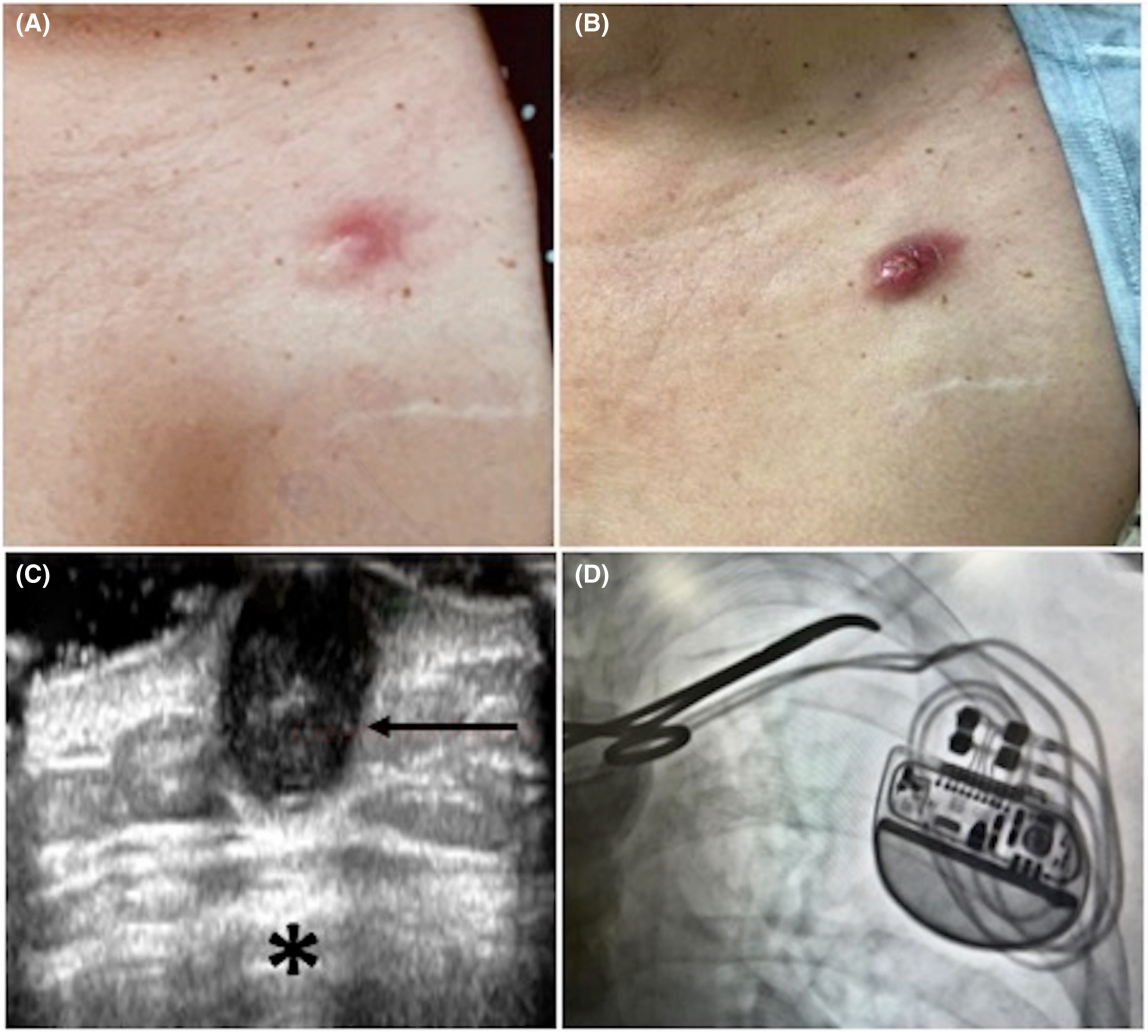
(A) A swollen, erythematous, and painful skin lesion at the upper part of the pacemaker pocket. (B) The lesion changed to a violaceous color and increased in size along with a superficial ulceration and fluid discharge. (C) An ultrasound scan of the skin lesion showed a hypoechoic and heterogeneous nodule with well‐defined borders located in the dermis and subcutaneous tissue (arrow). There was no compromise of deeper tissue and pacemaker pocket (asterisk). (D) Fluoroscopy showed that the skin lesion was over the upper part of the pocket where the leads run (under the Kelly forceps).

**FIGURE 2:**
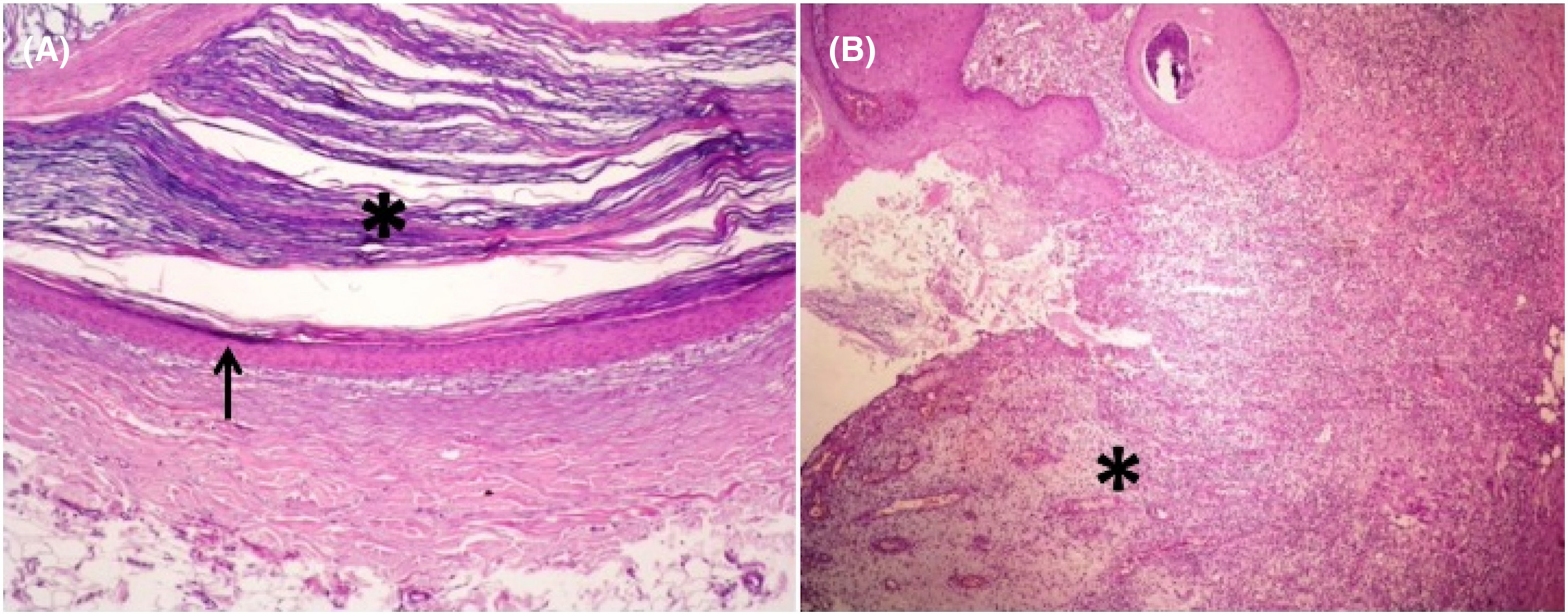
(A) Epidermal inclusion cyst. Skin fragment with cystic formation which is lined by epithelium epidermoid with a prominent granular layer (arrow) and filled with lamellated keratin material (asterisk) (H&E, 10×). (B) A fistulous tract with the development of granulation tissue (asterisk) (H&E, 10×).

A later cardiac implantable electronic device (CIED) infection could be secondary to bacteremia, or local factors such as inflammatory response or chronic trauma. An early diagnosis and treatment that involves antimicrobial therapy and complete device system removal are essentials in terms of morbidity and mortality. Nevertheless, some CIED infections can sometimes be difficult to diagnose and distinguish from other pathological processes. On the other hand, some inflammatory skin lesions may mimic CIED infections and their diagnosis are not always clear. Because of that, it is necessary to take a multidisciplinary approach in order to find a correct diagnosis. Also, complementary imagen tests (ultrasound or CT scan) can show if there is a compromise of the pacemaker pocket and define the extension of the lesion. In our case, an ultrasound scan was a useful tool to support a site revision with tissue samples sent to histopathological study instead of complete device system removal. Although pacemaker leads were initially suspected to be the primary site of infection, the surgical revision did not show compromise of them. Fortunately, the inflammatory skin lesion which was close to the pacemaker leads did not produce a secondary infection of them. We highlight the importance of taking a skin biopsy when unusual cutaneous findings are presented. The histopathological result can unravel the nature of the process.

The clinical presentation of a device‐related infection is often variable, ranging from subclinical device infections to a straightforward presentation with localized swelling, erythema, pain, device exposure, and purulent discharge along with fever and leukocytosis. This variable clinical setting can sometimes make the correct diagnosis difficult because other skin disorders can mimic a CIED infection. The differential diagnosis of CIED infections is: fluid collections (hematoma, pseudochyliform fluid), superficial infections (herpes zoster, cellulitis), allergic contact dermatitis, benign tumors (lipoma), and malignancies.^1^, ^2^, ^3^ As far as we know, EIC mimicking a pacemaker‐related infection has not been reported previously. EIC is an extremely rare condition that occurs secondary to implantation and proliferation of epidermal cells into the dermis and subcutaneous tissue because of injury such as surgery, puncture and trauma.^4^, ^5^ EIC has been reported after anesthetic infiltration, lumbar puncture, laparoscopic or abdominal surgery, and percutaneous procedures.^4^, ^5^ In our case, the trauma produced by puncture due to local anesthetic infiltration or during Seldinger technique for lead implantation can be the etiology of this particular EIC that can occur several years after surgery. The puncture could remove epidermal cells from epidermis or cells located inside the pocket during pocket creation to deeper tissues.

A multidisciplinary approach is required when a presumed CIED infection presents unusual cutaneous findings. Also, complementary imagen tests and histopathological study are necessary for unraveling the nature of the process. We highlight to keep in mind the differential diagnosis of a CIED infection in order to avoid unnecessarily complete device system removal.

## CONFLICT OF INTEREST

None of the authors have conflicts of interest to report regarding this manuscript.

## ETHICS APPROVAL STATEMENT

Not applicable.

## PATIENT CONSENT STATEMENT

The patient has given signed consent for publication of the case.

## CLINICAL TRIAL REGISTRATION

Not applicable.
